# Charter, Constitution, By-Laws, and Fee-Bill of the Eaton Medical Society, at Eaton, Ohio

**Published:** 1862-11

**Authors:** 


Charter, Constitution, By-Laws, and Fee-Bill of the Eaton Medical Society, at Eaton, Ohio.

We have received a copy of this pamphlet from Dr. R. Wallace, of Lewisburgh, Ohio; for which he has our sincere thanks.

